# Dietary patterns – A scoping review for Nordic Nutrition Recommendations 2023

**DOI:** 10.29219/fnr.v68.10541

**Published:** 2024-04-30

**Authors:** Henna Vepsäläinen, Jaana Lindström

**Affiliations:** 1 Department of Food and Nutrition, University of Helsinki, Helsinki, Finland; 2 Finnish Institute for Health and Welfare, Helsinki, Finland

**Keywords:** whole diet, eating habits, food consumption, healthy diet

## Abstract

**Background:**

A dietary pattern can be defined as the quantities, proportions, variety, or combination of foods and drinks typically consumed. The dietary pattern approach aims to place emphasis on the total diet as a long-term health determinant, instead of focussing on separate foods and nutrients, which may interact or confound each other.

**Aim:**

This scoping review describes the totality of evidence for the role of dietary patterns for health-related outcomes as a basis for setting and updating food-based dietary guidelines in the Nordic Nutrition Recommendations 2023 (NNR2023).

**Methods:**

We used evidence from 10 qualified systematic reviews identified by the NNR2023 project. No additional literature search was conducted.

**Results:**

Strong or moderate evidence linked dietary patterns high in vegetables, fruits, whole grains, fish, low-fat dairy and legumes, and low in red and processed meats, sugar-sweetened beverages, sugary foods- and refined grains with beneficial health outcomes, such as reduced risk of cardiovascular disease (CVD), type 2 diabetes, obesity, cancer, bone health, and premature death. We also found limited evidence suggesting a relationship with the described dietary patterns in childhood and decreased risk of obesity and hypertension later in life. Most studies have been conducted among adult populations, and thus, there is a need for studies in certain subgroups, such as children and adolescents as well as the elderly.

## Popular scientific summary

- Dietary pattern refers to the quantities, proportions, variety, or combination of foods and drinks typically consumed.
- A dietary pattern can be identified according to predefined indices, data-driven methods, or the distribution of nutrients.
- Dietary patterns in the Nordic and Baltic countries have been characterised as Western/sweet.
- There is moderate to strong evidence that dietary patterns high in vegetables, fruits, whole grains, nuts, legumes, low-fat dairy and seafood, and low in red and processed meat, refined grains, and sugar-sweetened foods and beverages are associated with decreased risk of all-cause mortality, CVD, obesity, type 2 diabetes, and breast and colorectal cancer.

Traditionally, research on the health effects of diet has focused on the relationships between nutrients, foods, or food groups, and health outcomes. However, as people eat foods and beverages in combinations, epidemiological studies have shifted their attention towards dietary patterns (1). A dietary pattern can be defined as the quantities, proportions, variety, or combination of foods and drinks a person typically consumes. Dietary patterns can be identified using predefined indices, which describe how closely a person’s diet resembles the given diet (2). For example, the Healthy Eating Index (HEI), the Mediterranean Diet Score (MedDiet Score), and the Healthy Nordic Diet have been developed to describe healthfulness of diets in different populations. The indices typically share similar characteristics, such as high consumption of fruits and vegetables, whole grains, fish, and unsaturated fats, and low consumption of sweets and sugar-sweetened beverages, but differ on some details because of cultural and food environmental differences between populations. For instance, the Mediterranean diet emphasises the use of olive oil (3), whereas the Healthy Nordic Diet recommends the use of rapeseed oil (4). The adherence to these dietary patterns is typically measured with a score, which can be interpreted to describe the healthfulness of the diet.

Another approach is to use data-driven methods, such as principal component analysis (PCA), factor analysis, or cluster analysis (5). These methods do not depend on the researcher’s definition of a healthful diet but are instead based on intercorrelations between food items (PCA, factor analysis) or individual differences in mean consumption (cluster analysis) (6). The data-driven methods describe the actual food consumption patterns in the given sample, and the patterns derived may be more or less healthy or unhealthy. A third approach to dietary patterns is to address the nutrient distribution of habitual diet, where distinction is made when, for example, at least one of the macronutrients (carbohydrates, fat, or protein) is outside the “acceptable macronutrient distribution range.” In the present paper, dietary patterns based solely on micronutrient intakes are not discussed, because the same micronutrients can be obtained from very different food sources, and thus, micronutrient intake distributions are not representative of food-level dietary patterns.

Dietary pattern analysis approach is advantageous since nutrients available in food may confound or interact with each other (1). Additionally, pattern analysis can be considered useful in evaluating dietary guidelines and may enable the detection of associations between diet and health outcomes, as the combined effect of an entire diet may be more powerful than the effects of its individual components (1, 2). Moreover, the whole-diet approach may be easier for the consumers to comprehend and implement in their everyday life. However, the dietary pattern approach also has its limitations. Most importantly, the identification of data-driven dietary patterns is strongly dependent on subjective and arbitrary decisions about combining of food groups and the labelling of dietary patterns (1). Furthermore, associated health outcomes may also be driven by single nutrients or foods included in the dietary pattern and not by the patterns in general, and thus, dietary pattern analysis should be considered as a complementary approach facilitating the understanding of diet-health-relationships (1). This scoping review summarises the evidence on the link between dietary patterns or whole-diet and health-related outcomes as a basis for setting and updating food-based dietary guidelines (FBDGs) in the Nordic Nutrition Recommendations (NNR) 2023 (Box 1). Both theory-based and data-driven dietary patterns were included.

*Box 1.* Background papers for Nordic Nutrition Recommendations 2023

- This paper is one of many scoping reviews commissioned as part of the Nordic Nutrition Recommendations 2023 (NNR2023) project (7).
- The papers are included in the extended NNR2023 report but, for transparency, these scoping reviews are also published in Food & Nutrition Research.
- The scoping reviews have been peer reviewed by independent experts in the research field according to the standard procedures of the journal.
- The scoping reviews have also been subjected to public consultations (see report to be published by the NNR2023 project).
- The NNR2023 committee has served as the editorial board.
- While these papers are a main fundament, the NNR2023 committee has the sole responsibility for setting dietary reference values in the NNR2023 project.

## Methods

The scoping review follows closely the protocol developed within the NNR2023 project (7). The sources of evidence used in the review follow the eligibility criteria described in an earlier paper (8). No *de novo* NNR2023 systematic reviews were conducted for the purposes of this paper, but altogether 10 qualified systematic reviews were recognised as suitable evidence (9). These qualified systematic reviews were used as primary evidence in this review.

## Diet intake in Nordic and Baltic countries

Most of the Nordic and Baltic countries monitor dietary intake among adult population, but dietary patterns are not routinely tracked, and the countries lack comprehensive, structured information on dietary patterns. Unlike predefined dietary indices, such as the Healthy Nordic Diet, data-driven dietary patterns, which describe the real food consumption patterns in a given population, can be used to describe dietary patterns on a general level. A few studies, examples of which are briefly described in the following paragraphs, have provided information about the dietary patterns in the Nordic and Baltic countries using predominantly PCA or factor analysis.

In general, it seems that “healthy” or “prudent” patterns characterised by frequent intake of for example vegetables, fruits, high-fibre bread, and vegetable oils have been identified in Swedish (10–14), Norwegian (13, 15, 16), Danish (13, 17), and Finnish (14, 18) adult populations. In addition, a “traditional” or “mixed” dietary pattern characterised by frequent consumption of for example potatoes, meat, bread, and fish has shown to be evident in Sweden (10, 11, 14), Norway (13, 15), Denmark (13), and Finland (14, 18). The “traditional” and “mixed” dietary patterns usually share traits of both healthy and unhealthy diets, and thus, they cannot be easily categorised as healthy or unhealthy.

A frequently identified dietary pattern in the Nordic countries is also a “Western” or a “sweet” dietary pattern, which consists of foods typically considered unhealthy, such as fried potatoes and fast food, meat products, sweets, and soft drinks (11–13, 15, 16, 18). Interestingly, some studies have also identified an “alcohol” pattern, which seems to be characterised by frequent consumption of, for example, meat products and salty snacks, on top of alcohol usage (14, 16, 17). In a few studies, alcohol consumption has been associated with an unhealthy dietary pattern (12, 13, 18), but it has also been shown to relate to a traditional pattern among Norwegian Sami population (15) and to a prudent pattern among Finnish middle-aged men (18). When analysed separately, similar patterns seem to emerge both among men and women (e.g. “healthy” or “traditional”) (10, 12), but in general women tend to score higher or be over-represented in the “healthy” patterns (11, 15, 17).

Studies among children and adolescents have yielded rather similar patterns apart from the alcohol-related dietary patterns. A “healthy” pattern seems to be most prominent and has been identified in Finnish (19, 20), Icelandic (21), Estonian, and Swedish (22) populations. In addition, “unhealthy” (19, 20, 22) and “traditional” or “mixed” (19–21) patterns have also been found among children in the Nordic and Baltic countries.

## Health outcomes relevant for Nordic and Baltic countries

### Obesity

In adults, moderate evidence from 54 longitudinal studies showed an association between favourable outcomes related to body weight (e.g. lower body mass index [BMI], waist circumference) or risk of obesity and dietary patterns characterised by higher intakes of vegetables, fruits, whole grains, seafood, and legumes; moderate intakes of dairy products (with emphasis on low- and non-fat dairy) and alcohol; lower intakes of meats (including red and processed meats); and low intakes of sugar-sweetened foods and beverages and refined grains (23). The foods included in the beneficial patterns contain higher levels of unsaturated fats and lower levels of saturated fats, cholesterol, and sodium. A systematic review of 12 longitudinal studies concluded that there is limited evidence linking dietary patterns lower in fruits, vegetables, whole grains, and low-fat dairy and higher in added sugars, refined grains, fried potatoes, and processed meats with higher BMI in adolescence (23). The 2020 Dietary Guidelines Advisory Committee (USA) could not evaluate the possible associations between diets based on macronutrient distribution and obesity because of insufficient evidence.

### Cardiovascular diseases

Altogether 149 articles examining dietary patterns and risk of CVDs among adults were included in a systematic review published by the 2020 Dietary Guidelines Advisory Committee (24). The authors concluded that strong and consistent evidence links decreased risk of CVD with dietary patterns characterised by higher consumption of vegetables, fruits, whole grains, low-fat dairy, and seafood and lower consumption of red and processed meat, refined grains, and sugar-sweetened foods and beverages. Most studies have also shown regular consumption of nuts and legumes and moderate consumption of alcohol to be beneficial in reducing CVD risk. A systematic review of four longitudinal studies provided limited evidence showing that lower blood pressure and blood lipid levels later in life associated with dietary patterns higher in vegetables, fruits, whole grains, fish, low-fat dairy and legumes, and lower in sugar-sweetened beverages, other sweets, and processed meat among children and adolescents (24). Non-energy restricted diets based on macronutrient distribution with either carbohydrate, fat, and/or protein proportion outside of the “acceptable macronutrient distribution range” were judged to be neither beneficial nor detrimental among adults, albeit the evidence was limited because of, for example, limitations in the study designs. No studies investigating CVDs and diets based on macronutrient distribution consumed by children were identified.

### Type 2 diabetes

The systematic review (25) identified only one article exploring (retrospectively) the association between dietary patterns in adolescence and risk of type 2 diabetes by middle age. The evidence to give recommendations was considered inadequate. As regards to adults, 52 articles were identified in the literature search covering years 2014 to 2020. The authors concluded that there is moderate evidence to indicate that dietary patterns higher in vegetables, fruits and whole grains, and lower in red and processed meats, high-fat dairy products, refined grains and sweets/sugar-sweetened beverages reduce the risk of developing type 2 diabetes. The authors identified 23 articles (2 randomised clinical trials (RCTs), 21 prospective cohort studies) published between 2000 and 2020 where diets with differing macronutrient distributions were studied. Most of the studies explored diet with proportion of carbohydrates below and fat above the acceptable range. The authors concluded that it is not possible to make recommendations about macronutrient distribution and risk of type 2 diabetes based on the available evidence.

### Cancer

In a systematic review of 26 studies (3 RCTs, 21 prospective cohorts, and 2 nested case-control studies), dietary patterns characterised by high intakes of vegetables, fruits and whole grains, and low intakes of animal-source foods and refined carbohydrates were linked to reduced risk of postmenopausal breast cancer (26). The evidence was graded moderate. The evidence regarding dietary patterns and premenopausal breast cancer risk was limited but parallel.

By systematically reviewing 24 studies (2 RCTs, 21 prospective cohorts, and 1 nested case-control study), the 2020 Dietary Guidelines Advisory Committee concluded that moderate evidence links dietary patterns higher in vegetables, fruits, legumes, whole grains, lean meats and seafood, and low-fat dairy, and low in red and processed meats, saturated fat, sugar-sweetened beverages, and sweets with lower risk of colon and rectal cancer (26). Moderate evidence showed that dietary patterns higher in red and processed meats, French fries, potatoes, and sources of sugars (e.g. sugar-sweetened beverages, sweets and desserts) associated with a greater colon and rectal cancer risk.

Regarding lung cancer, the 2020 Dietary Guidelines Advisory Committee reviewed seven prospective cohort studies and one nested case-control study (26). The authors concluded that limited evidence shows a relationship between dietary patterns higher in vegetables, fruits, seafood, grains and cereals, legumes and lean meats, and lower in fat or non-fat dairy products with lower risk of lung cancer. The association was evident primarily among former and current smokers. A systematic review of seven prospective cohort studies and one nested case-control study yielded limited evidence suggesting no relationship between dietary patterns and risk of prostate cancer (26).

### Bone health

In a systematic review of nine prospective cohort studies, dietary patterns characterised by high consumption of fruits, vegetables, legumes, nuts, low-fat dairy, whole grains and fish, and lower consumption of meats (particularly processed meats), sugar-sweetened beverages, and sweets was linked to beneficial bone health outcomes in adults (27). The main bone health outcome used in the studies was hip fractures, and the evidence was graded moderate. Only two articles examining the link between dietary patterns and bone health outcomes in adolescents were identified. Thus, the relationship between dietary patterns in adolescence and later bone health could not be estimated.

### Neurocognitive health

The 2020 Dietary Guidelines Advisory Committee conducted a systematic literature search to identify studies evaluating the association between dietary patterns and neurocognitive health (28). Altogether 26 studies (4 RCTs, 22 observational studies) using cognitive performance, age-related cognitive decline, mild cognitive impairment and/or incident dementia as outcome and published between January 2014 and February 2020 were identified. In the studies, both exposures and outcomes were measured using various approaches. The conclusion was that there is limited evidence suggesting that dietary patterns containing vegetables, fruits, unsaturated vegetable oils and/or nuts, legumes, and fish or seafood consumed during adulthood are associated with lower risk of age-related cognitive impairment and/or dementia.

### Other health outcomes

The 2020 Dietary Guidelines Advisory Committee systematically reviewed four prospective cohort studies examining the association between dietary patterns and sarcopenia (29). However, because of methodological inconsistencies and risk of bias, the authors concluded the evidence to be insufficient to determine the relationship between dietary patterns and sarcopenia.

A total of 26 articles (mostly from prospective cohort studies) were included in a systematic review investigating the association between dietary patterns during pregnancy and risk of excessive gestational weight gain during pregnancy (30). The review concluded that limited evidence links dietary patterns higher in vegetables, fruits, nuts, legumes and fish, and lower in added sugar, and red and processed meat with lower risk of excessive gestational weight gain during pregnancy. Strong conclusions could not be drawn because of lack of RCTs and controlling for key confounders, risk of bias, and unrepresentativeness of the samples.

In a systematic review of three RCTs and two cross-sectional studies, the 2020 Dietary Guidelines Advisory Committee concluded that limited evidence supports the finding that maternal dietary patterns associate with the relative proportions of saturated fat, monounsaturated fatty acids, and polyunsaturated fatty acids in human milk (31). However, because of the small number of studies included and methodological differences in defining dietary patterns, the characteristics of beneficial dietary patterns could not be unambiguously described. In addition, the included studies had small and unrepresentative samples, did not control for key confounders, and were heterogeneous in terms of timing and methods of human milk collection. With regards to other milk composition parameters and quantity, no evidence was available.

### Mortality

The 2020 Dietary Guidelines Advisory Committee reported in a systematic review consisting mostly of prospective cohort studies (140 studies out of the 141 included studies) that dietary patterns characterised by frequent consumption of vegetables, fruits, legumes, nuts, whole grains, unsaturated vegetable oils, and fish, lean meat, or poultry, when meat was included, associated with decreased risk of all-cause mortality (32). The patterns contained relatively low amounts of red and processed meat, high-fat dairy, and refined carbohydrates or sweets. The evidence was ranked strong and demonstrated within adult and older adult populations. The link between diets based on macronutrient distribution and all-cause mortality could not be assessed because of insufficient evidence.

## Mechanisms

The recommended dietary patterns are typically nutrient-dense and include a variety of food groups that have been found to associate with health benefits. For example, consumption of fruit, vegetables, and wholegrain cereals have been linked to reduced risk of CVD, cancer, and all-cause mortality (33, 34). Both fruits and vegetables as well as wholegrain products contain a wide range of nutrients and phytochemicals, such as fibre, vitamin C, carotenoids, potassium, antioxidants, flavonoids, and other compounds, which can act to reduce the risk of chronic diseases through several biological mechanisms. These mechanisms can include, for example, increased satiety (35), modulation of stress- and defence-related gene expression (36), promotion of beneficial microbial metabolites (37), or reduction of low-grade inflammation (38). Additionally, frequent fish consumption has also been linked to health benefits (39). Fatty fish is a significant source of omega-3 fatty acids, especially eicosapentaenoic acid (EPA) and docosahexaenoic acid (DHA), which have been shown to, for example, prevent inflammation and decrease triglyceride concentration through several mechanisms including alterations in cell membrane composition and eicosanoid production (40).

The recommended dietary patterns usually include restricted amounts of processed or red meat and sugar-sweetened beverages. High consumptions of processed and red meat have been linked to increased risk of cancer, type 2 diabetes, and mortality (41). The mechanism linking processed meat consumption with health outcomes remains unclear, but for example high levels of sodium (42), polycyclic aromatic hydrocarbons, and heterocyclic aromatic amines as well as nitrosyl-heme have been hypothesised to play a role (43). High intake of sugar-sweetened beverages has been shown to associate with weight gain and obesity (44) through increased energy intake (45). Sugar-sweetened beverage consumption may also link to other adverse health outcomes, such as greater incidence of type 2 diabetes (46). High consumption of sugar-sweetened beverages can also associate with decreased bone health through multiple potential mechanisms including sugar, phosphate, acidity, and caffeine, which can all negatively affect bone metabolism (47). Dietary patterns that include large amounts of energy-dense foods with low nutrient density (e.g. sugar-sweetened beverages and other foods high in sugar, sodium, and/or saturated fat) may also lead to micronutrient deficiencies, which in turn can predispose to chronic diseases.

To sum up, multiple mechanisms mediating the beneficial and harmful effects of food groups, foods, and nutrients have been identified and probably contribute to the health effects of dietary patterns. Moreover, nutrients are ingested as a part of the food matrix and composite meals, which may modulate their absorption and functions in the body. Nutrients and other compounds in the diet may have synergistic, antagonistic, or other interactive effects as part of the whole diet. Some of these effects remain unknown, and thus, they cannot be measured or controlled for.

### Food-based dietary guidelines

In general, dietary patterns higher in vegetables, fruits, whole grains, fish, low-fat dairy and legumes, and lower in red and processed meats, sugar-sweetened beverages, sugary foods (e.g. sweets), and refined grains were found to be protective in terms of CVD, type 2 diabetes, obesity, breast and colon cancer, bone health, and premature death among adult populations. The evidence was graded as strong or moderate. Limited evidence was found for the protective effect of the aforementioned dietary patterns regarding lung and prostate cancer, neurocognitive health, and gestational weight gain. Similarly, limited evidence linked the described dietary patterns in childhood and/or adolescence with smaller risk of obesity and hypertension later in life. It was not possible to make recommendations about macronutrient distribution and risk of chronic diseases based on the available evidence.

The beneficial dietary patterns are characterised by food items that have been linked to improved health outcomes, such as vegetables, fruits, whole grains, and legumes. These foods are in general high in micronutrients and contain low or moderate amounts of energy. Similarly, the patterns typically contain only small amounts of food items generally linked with unbeneficial health outcomes, such as red and processed meat and sugar-sweetened beverages. Red and processed meat may link to poor health via various pathways, whereas sugar-sweetened beverages contain virtually no micronutrients but may contribute to excess energy intake. Excess energy intake will, in turn, lead to obesity, which is an important risk factor for cardiometabolic diseases and several types of cancer (48).

Dietary fat quality can contribute to the risk of several chronic diseases, as mentioned earlier. In general, the foods included in the beneficial patterns contain higher levels of unsaturated and lower levels of saturated fats. Specifically fish, which is a good source of unsaturated fatty acids, was included in the healthy dietary pattern in many of the qualified systematic reviews. Inclusion of vegetable oils with high unsaturated and low saturated fat content in recommended dietary patterns could further improve dietary fat quality.

Dietary patterns higher in plant-based foods and lower in animal-based foods are environmentally more sustainable than patterns high in animal-based foods (49). As the pattern identified in this scoping review emphasises plant-based foods (vegetables, fruits, whole grains), it can be argued that compared to current diets in the Nordic and Baltic countries, the recommended pattern would be beneficial for public health and environmental sustainability. However, the recommended pattern also includes animal-based foods, such as fish and low-fat dairy products. It is worth noticing that dietary patterns are very broad descriptions of diet and cannot be interpreted as exact recommendations on the composition of a healthy diet. These findings do not indicate that a dietary pattern must contain animal-based foods to be healthy, nor do they show that low levels of meat consumption would be disadvantageous.

The methods used to derive dietary patterns are mainly designed to capture the existing diets of the populations, which typically contain both plant- and animal-based foods. Thus, based on the evidence presented by the qualified systematic reviews, conclusions on the healthiness of vegan or vegetarian diets as opposed to mainly plant-based diets that may also contain fish, low-fat dairy products, and low amounts of meat and/or other animal-based foods (the recommended diet) cannot be drawn. As for example in Finland, the prevalence of vegetarianism has increased from 0.7 to 1.8% between 2012 and 2017 (50) and may continue to rise, future studies should aim to examine dietary patterns among vegans and vegetarians and compare their health effects with those of a healthy, mixed dietary pattern.

Many of the qualified systematic reviews used as sources of evidence in this scoping review were able to identify studies in both adult and child populations. However, the evidence regarding the associations between childhood dietary patterns and later health outcomes were mostly limited because of the small number of prospective studies. Thus, more longitudinal studies tracking dietary patterns and health outcomes from childhood are needed. Moreover, studies investigating osteoporosis and sarcopenia were very few, and more studies are also needed among the elderly. It is noteworthy that the majority of the studies included in the qualified systematic reviews were observational, which limits the conclusions. However, as the considered health outcomes take multiple years to develop, well-designed, good-quality prospective studies are in many cases the best possible evidence.

The scoping review used 10 qualified systematic reviews identified by an initial literature search as sources of evidence. The qualified systematic reviews were conducted by the United States Department of Agriculture (USDA) and were of good quality. However, most of them covered the timeframe up until 2019. Thus, studies published in 2019–2022 have not been covered. It is unlikely that during that time, such a large body of conflicting evidence would have accumulated that the strong or moderate evidence had to be downgraded into limited. By contrast, it is possible that some of the limited evidence would now be graded as moderate.

As mentioned in the introduction, because of the complex associations between nutrients, foods, diets, and health outcomes, the dietary pattern analysis should be considered as a complementary approach when examining the relations between diet and health (1). Furthermore, as dietary assessment methods and food groups included in the analysis influence the results, comparison of dietary patterns between study populations is challenging. Thus, the method of constructing the dietary patterns may lead to unspecific and sometimes even partly incorrect conclusions. To create more specific knowledge on dietary patterns, future studies should consider focusing on more detailed food groups, such as subtypes of vegetables, fried and boiled/mashed potatoes, red meat and processed meat, or different types of dairy products. However, as dietary patterns reflect the whole diet of the participants rather than the intake of specific foods or nutrients, the level of detail in analysis cannot be similar to studies investigating the associations between single nutrients or foods and health outcomes.
